# Development of a Potent Stabilizer for Long-Term Storage of Foot-and-Mouth Disease Vaccine Antigens

**DOI:** 10.3390/vaccines9030252

**Published:** 2021-03-12

**Authors:** Ah-Young Kim, Hyejin Kim, Sun Young Park, Sang Hyun Park, Jae-Seok Kim, Jung-Won Park, Jong-Hyeon Park, Young-Joon Ko

**Affiliations:** Animal and Plant Quarantine Agency, Gimcheon 39660, Gyeonsangbuk-do, Korea; mochsha@korea.kr (A.-Y.K.); hyejin86@korea.kr (H.K.); sun3730@korea.kr (S.Y.P.); shpark0205@korea.kr (S.H.P.); kimjs0728@korea.kr (J.-S.K.); parkjw6254@korea.kr (J.-W.P.); parkjhvet@korea.kr (J.-H.P.)

**Keywords:** foot-and-mouth disease (FMD), buffer, excipient, stabilizer, virus, vaccine antigen

## Abstract

A local virus isolate, O/SKR/JC/2014 (O JC), has been considered as a candidate vaccine strain in the development of a domestic foot-and-mouth disease (FMD) vaccine in Korea. However, producing and preserving a sufficient quantity of intact vaccine antigens from the O JC strain was difficult owing to its distinctive structural instability compared to other candidate vaccine strains. Based on this feature, the O JC strain was adopted as a model virus for the stabilization study to determine the optimal stabilizer composition, which enables long-term storage of the FMD vaccine antigen in both aqueous and frozen phases. In contrast to O JC vaccine antigens stored in routinely used Tris-buffered or phosphate-buffered saline, those stored in Tris-KCl buffer showed extended shelf-life at both 4 °C and −70 °C. Additionally, the combined application of 10% sucrose and 5% lactalbumin hydrolysate could protect O JC 146S particles from massive structural breakdown in an aqueous state for up to one year. The stabilizer composition was also effective for other FMDV strains, including serotypes A and Asia 1. With this stabilizer composition, FMD vaccine antigens could be flexibly preserved during the general production process, pending status under refrigeration and banking under ultrafreezing.

## 1. Introduction

Foot-and-mouth disease (FMD) caused by the FMD virus (FMDV), is a highly contagious vesicular disease of cloven-hoofed animals [1]. To prevent its outbreak, many countries are vaccinating susceptible farm animals such as cattle and pigs [2].

The FMD vaccine is mainly comprised of inactivated virus particles and adjuvants. Intact virus particle, known as 146S particle owing to its sedimentation coefficient, is composed of 60 copies of a protomer. Five protomers, each of which consists of one set of structural proteins (VP1, VP2, VP3, and VP4), assemble to form a pentamer, and 12 copies of the pentamer and the encapsidated viral RNA form a 146S particle [3]. Intact virus particles, followed by empty capsids (75S) are known to confer the most potent protective immunity to vaccinated animals compared to pentamers (12S) that are poor at conferring immune protection [4]; however, 146S can easily dissociate into less immunogenic 12S particles by weak acid or mild heat [4,5]. Therefore, stabilization of 146S particles is a key technique for FMD vaccine production.

Thus, many previous studies have focused on the methods of generating thermostable or acid-resistant FMDV 146S particles by genetically manipulating specific sequences encoding viral structure proteins [6,7,8]. However, structural stabilization of virus particles by genetic modification can paradoxically hamper virus uncoating and replication. Moreover, this technique cannot be used as a solution for vaccine antigen production in wild-type FMDV. Instead, a strategy to stabilize FMD vaccine antigens by suspending them in proper buffers with additives was sought.

Previous studies have revealed that FMDV capsid stability varies depending on buffers containing various salts at different concentrations [9,10,11]. In addition, several classes of additives have been proven to be effective for the stabilization of viruses, including FMDV [9,10,11,12,13]. Although the aforementioned studies showed some degree of elevation of the virus stability, it was uncertain whether those compositions could genuinely prolong the shelf-life of FMD vaccine antigens in the aqueous phase for months or protect the antigens from freeze–thaw damage.

During the general vaccine production process, vaccine antigens exist in a suspended state, and if needed, should be stored for days to weeks before final formulation with adjuvants, depending on the manufacturer’s batch production system. Furthermore, vaccine antigens can be stored in a frozen state for longer storage or kept in antigen banks. As FMDV is particularly notorious for its instability [14], the 146S antigen stabilization technique during the production process is unknown and protected as intellectual property by commercial vaccine companies. Herein, we introduced a highly unstable type O strain isolated in Korea as a useful model virus for the stabilization study and aimed to provide a practical buffer and excipient composition enabling the long-term storage of FMD vaccine antigen in both aqueous and frozen phases.

## 2. Materials and Methods

### 2.1. Production of Vaccine Antigens

Four strains of FMDV were used in this study. FMDV O SKR/JC/2014 (O JC), O SKR/BE/2017 (O BE), and A SKR/YC/2017 (A YC) strains were Korean isolates, while FMDV Asia 1 Shamir/ISR/1989 (As 1 Shamir) was of foreign origin. Each strain of FMDV was inoculated in BHK21 suspension cells at a multiplicity of infection (MOI) of 0.002 and was incubated at 37 °C in a 5% CO_2_ shaking incubator at 110 rpm. Subsequently, the clarified virus culture supernatant was harvested via centrifugation (4000× *g*, 20 min) at 16 h post-infection.

Then, viruses were inactivated by the addition of 3 mM binary-ethylenimine (BEI) (Sigma-Aldrich, St. Louis, MO, USA) and incubated in a shaking incubator at 26 °C for 24 h [15]. Residual BEI was quenched using 2% sodium thiosulfate (Daejung Chemicals, Siheung-si, Korea). The inactivated FMDV culture supernatant was concentrated by mixing it with a final concentration of 7.5% (*w*/*v*) polyethylene glycol (PEG) 6000 (Sigma-Aldrich) and 0.5 M NaCl (Sigma-Aldrich). The precipitate was obtained by centrifugation (10,000× *g* for 30 min) and further purified by sucrose gradient ultracentrifugation as described in a previous study [16]. The purified 146S antigen was then resuspended in each buffer and excipient composition.

### 2.2. Buffers and Excipients

The basic composition of the Tris-buffered saline (TBS) was 50 mM Tris-HCl and 100 mM NaCl (pH 7.6), while that of Tris-KCl (TK) was 20 mM Tris-HCl and 300 mM KCl (pH 7.6). Phosphate-buffered saline (PBS; Corning, Manassas, VA, USA), which was composed of 154 mM NaCl, 5.6 mM Na_2_HPO_4_, and 1 mM KH_2_PO_4_ (pH 7.6), was used alone or supplemented using 150 mM NaCl and 50 mM MgCl2 (PNM buffer, pH 7.6). TNE buffer was prepared by adding 1 mM ethylenediaminetetraacetic acid to the TBS (pH 7.6), and KP buffer was composed of 600 mM potassium phosphate and 500 mM KCl (pH 7.6). For the combinational excipients, sucrose (Sigma-Aldrich) and lactalbumin hydrolysate (Sigma-Aldrich) were used as indicated % (*w*/*v*) concentrations.

### 2.3. S particle Quantification

The quantification of 146S particles was performed using either sucrose density gradient (SDG) fractionation or size-exclusion high-performance liquid chromatography (SE-HPLC). For the SDG fractionation, the sample solution was layered onto 15–45% sucrose density gradients and ultra-centrifuged again at 100,000× *g* for 4 h at 4 °C using an SW41Ti rotor. The ultra-centrifuged SDG was fractionated using a continuous density gradient fractionator (Teledyne ISCO, Lincoln, NE, USA), and the absorbance of each fraction at 254 nm was recorded using a spectrophotometer component of the instrument. The area under the peak for specific fractions was measured to calculate the quantity of 146S antigens (µg/mL) according to a previous study [17]. In the case of SE-HPLC, the analysis was performed on a TSKgel G4000PWXL (300 mm × 7.8 mm I.D.) column (TOSOH Bioscience, Tokyo, Japan) combined with a TSKgel PWXL Guardcol (40 mm × 6.0 mm I.D.) guard column (TOSOH Bioscience, Tokyo, Japan) using an Agilent 1260 Infinity II system (Agilent Technologies, Santa Clara, CA, USA), composed of a quaternary pump with an online degasser, autosampler with a sample cooler, a thermostatic column compartment, and a variable wavelength detector operating at 254 nm. Samples were pretreated using benzonase (Merck, Darmstadt, Germany) before analysis to digest host cell-derived DNA, as described in a previous study [18]. The mobile phase was composed of 30 mM Tris-HCl and 400 mM NaCl (pH 8.0), and the flow rate was set at 0.5 mL/min. The area under the target peak was integrated using the OpenLAB CDS ChemStation software, and the quantity of 146S antigens (µg/mL) was calculated according to a previous study [18].

### 2.4. Stability Tests

To compare the stability of the FMDV strains, 20 µg/mL of 146S antigens of each strain, suspended in TBS (pH 7.6), was heated at 45 °C, and samples were collected at 1, 2, 5, 15, and 30 min after heating. For the long-term storage test, FMDV O JC vaccine antigens were suspended in each buffer and excipient composition and stored as liquid either at 4 °C or −70 °C for up to 1 year. The percentage of recovery was calculated at each time point as follows: 146S antigen content after storage or heating/146S antigen content before storage or heating × 100.

Accelerated stability tests were conducted by heating at 50 °C for 30 min to validate the effectiveness of the selected buffer and excipient composition to other FMDV strains in addition to O JC. Relative recovery (%) was calculated from the remaining content of the 146S antigen after heating when the antigen content of 10% sucrose with 5% lactalbumin hydrolysate (SLA) in TK was set to 100%.

### 2.5. Transmission Electron Microscopy

The morphology of the intact FMDV particles was observed using transmission electron microscopy (TEM). Vaccine antigens stored in each test composition after PEG concentration were layered on top of sucrose gradients and ultra-centrifuged at 100,000× *g* for 4 h. The band between the 30% and 35% sucrose layers was collected and ultra-centrifuged at 100,000× *g* for 4 h. The resulting pellet was dialyzed using TK buffer (pH 7.6) to eliminate residual sucrose at 4 °C. One drop of the purified FMDV suspension was placed on formvar-coated grids and negatively stained with 1% uranyl acetate. The FMDV particles were examined using TEM (Hitachi 7100; Hitachi, Tokyo, Japan).

### 2.6. Statistical Analysis

Unless otherwise stated, all values were presented as mean ± standard error of the mean. All experiments were performed in triplicate. Statistical analyses were performed using one-way analysis of variance (ANOVA) and two-way repeated measurement ANOVA followed by paired t-test with Bonferroni correction as post hoc analysis for multiple comparisons using SPSS Statistics version 26.0. software (IBM Corp., Armonk, NY, USA). Statistical significance was set at *p* < 0.05, or was defined as *, *p* < 0.05, or **, *p* < 0.01.

## 3. Results

### 3.1. Identification of FMDV O/SKR/JC/2014 (O JC) as a Highly Unstable Model Virus

**Figure 1:**
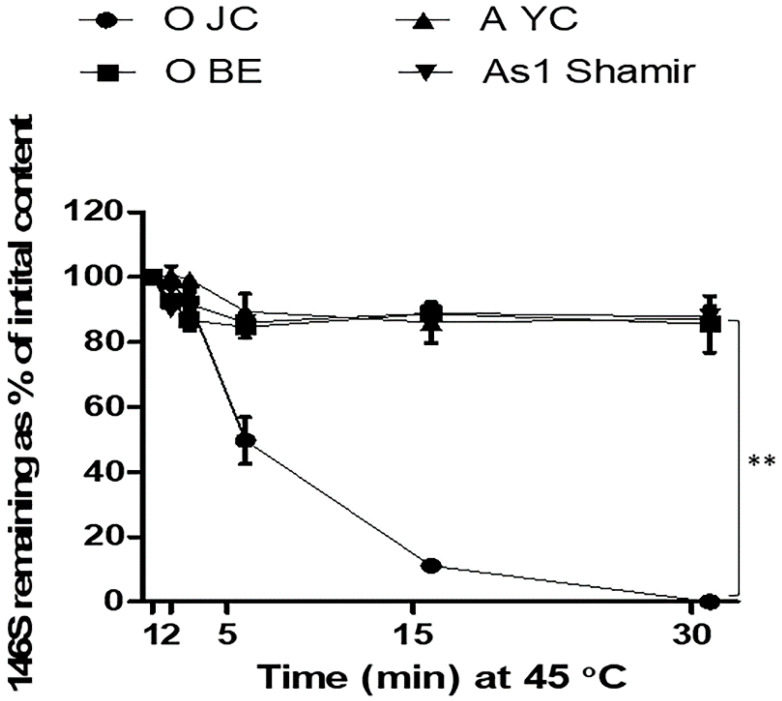
Characteristic thermal instability of foot-and-mouth disease virus (FMDV) O SKR/JC/2014. FMDV O SKR/JC/2014 (O JC), O SKR/BE/2017 (O BE), A SKR/YC/2017 (A YC), and Asia 1 Shamir/ISR/1989 (As 1 Shamir) were heated at 45 °C and their 146S antigen recovery (%) was calculated at each time point (1, 2, 5, 15, and 30 min) based on the antigen quantity measured by sucrose density gradient fractionation. **, *p* < 0.01.

When the four strains of FMDV, including O JC, O BE, A YC, and As 1 Shamir, were suspended in normal TBS (pH 7.6) and subjected to heating at 45 °C for 30 min, all the other strains, except for O JC, showed a gentle decrease in 146S content as their loss after 30 min was less than 20% (Figure 1). However, O JC displayed a drastic decrease, as 50% of the initial 146S content was lost after 5 min and was completely dissociated after 30 min (Figure 1). Thus, it was identified that FMDV O JC has characteristic instability compared to other FMDV strains.

### 3.2. Screening of Buffers during Short-Term Storage of FMDV O JC Vaccine Antigen

**Figure 2:**
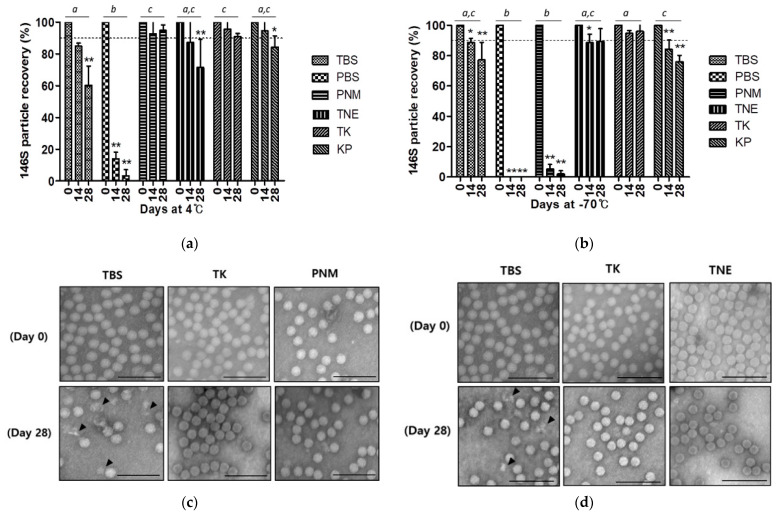
Effectivity of buffers for short-term storage of FMDV O JC vaccine antigen. (**a**) The 146S particle recovery (%) in each buffer during 4-week storage at 4 °C; (**b**) The 146S particle recovery (%) in each buffer during 4-week storage at −70 °C. The dotted line is set at 90% recovery. *, *p* < 0.05, **, *p* < 0.01. Different lowercase letters indicate significant differences at *p* < 0.05; (**c**) Microscopic observation of 146S particles stored in the aqueous phase; (**d**) Microscopic observation of 146S particles stored in frozen phase. Scale bar = 100 μm. Arrowheads indicate degrading particles. Abbreviations: TBS, Tris-buffered saline (pH 7.6); PBS, phosphate-buffered saline (pH 7.6); PNM, PBS supplemented with NaCl and MgCl2 (pH 7.6); TNE, TBS supplemented with ethylenediaminetetraacetic acid (pH 7.6); TK, Tris-KCl buffer (pH 7.6); KP, potassium phosphate buffer (pH 7.6).

FMDV O JC was suspended in each buffer and stored at either 4 °C or −70 °C to assess the protective capacity of commonly used buffers against viral antigen dissociation. In both aqueous and frozen states, PBS showed the most drastic reduction in the O JC vaccine antigen after 4-week storage. While PNM was the best buffer for vaccine antigens in the aqueous phase, it did not work in the frozen phase. In addition, TNE appeared to be good as a stabilizing buffer in the frozen phase; however, its preserving capacity was not that high in the aqueous phase. TBS and KP exhibited moderate preservation in both phases. TK was the only buffer that could prevent the loss of vaccine antigen by less than 10% after 4 weeks of storage in both phases (Figure 2a,b). With TBS as a control, samples from the two best buffers in each phase were observed by TEM. Unlike TBS, which showed degrading viral particles at both temperature conditions, TK and another selected buffer at each temperature rarely showed any morphological abnormality (Figure 2c,d).

### 3.3. Effectivity of Candidate Buffers for Long-Term Storage of FMDV O JC Vaccine Antigen

**Figure 3:**
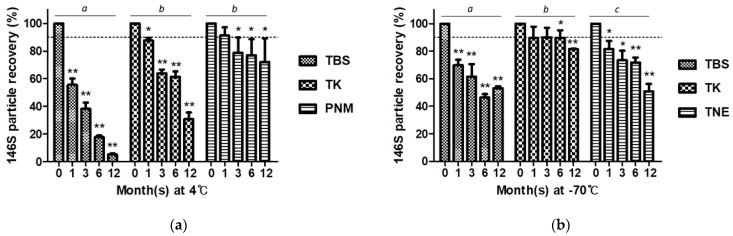
Effectivity of buffers for long-term storage of FMDV O JC vaccine antigen. (**a**) The 146S particle recovery (%) in each buffer during 1-year storage at 4 °C; (**b**) The 146S particle recovery (%) in each buffer during 1-year storage at −70 °C. The dotted line is set at 90% recovery. *, *p* < 0.05, **, *p* < 0.01. Different lowercase letters indicate significant differences at *p* < 0.05.

The two best buffers in each phase that were identified as effective for short-term storage and TBS as control were assessed for long-term storage up to 1 year. In the aqueous state, no buffer effectively preserved the FMDV O JC vaccine antigen after 3 months (Figure 3a). Similarly, no buffer could maintain the FMDV O JC recovery up to 90% in the frozen state after a year, although the 146S recovery of TK did not show a significant reduction until 6 months and was 81.41 ± 0.29% at 12 months (Figure 3b).

### 3.4. Effectivity of the Combinational Use of a Selected Buffer and Excipients

**Figure 4:**
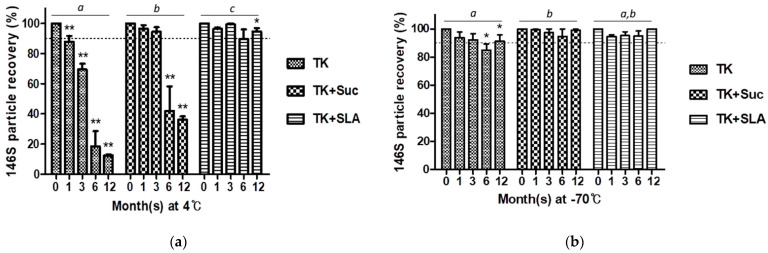
Effectivity of excipients for long-term storage of FMDV O JC vaccine antigen. (**a**) The 146S particle recovery (%) in each buffer during 1-year storage at 4 °C; (**b**) The 146S particle recovery (%) in each buffer during 1-year storage at −70 °C. The dotted line is set at 90% recovery. *, *p* < 0.05, **, *p* < 0.01. Different lowercase letters indicate significant differences at *p* < 0.05. Abbreviations: TK, Tris-KCl buffer (pH 7.6); Suc, 20% sucrose; SLA, 10% sucrose with 5% lactalbumin hydrolysate.

Based on a preliminary screening among several kinds of excipient composition, 20% sucrose (Suc) and SLA that were known to be effective in FMDV stabilization were selected for the long-term preservation test with TK buffer (data not shown). In contrast to the sole use of TK, the combined use of either excipient composition enhanced the stability of the O JC vaccine antigen in the aqueous phase. Particularly, SLA in TK showed dramatic improvement as it displayed more than 90% of antigen preservation until 12 months at 4 °C (Figure 4a). Meanwhile, the mild reduction in antigen recovery, shown in the sole use of TK in the frozen phase, was not observed when either Suc or SLA was used as excipients in the TK (Figure 4b).

### 3.5. Compatibility of the Candidate Stabilizer Composition with Other FMDV Strains

**Figure 5:**
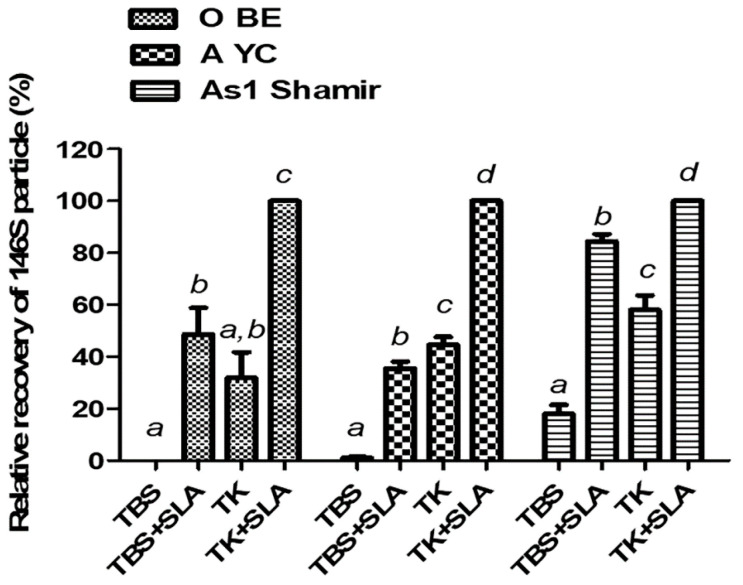
Compatibility of the candidate stabilizer composition with other FMDV strains. Relative recovery (%) was calculated from the remaining content of the 146S antigen after heating at 50 °C for 30 min when the antigen content of SLA in TK is set as 100%. Different lowercase letters indicate significant differences at *p* < 0.05. Abbreviations: O BE, O SKR/BE/2017; A YC, A SKR/YC/2017; As 1 Shamir, Asia 1 Shamir/ISR/1989; TBS, Tris-buffered saline (pH 7.6); TK, Tris-KCl buffer (pH 7.6); SLA, 10% sucrose with 5% lactalbumin hydrolysate.

To identify whether the stabilizing effect of SLA in TK that was revealed in the long-term storage of FMDV O JC is compatible with other FMDV strains, an accelerated stability test was conducted by heating the 146S antigens of each strain, suspended in TBS (pH 7.6), at 45 °C for 30 min. Regardless of the serotype, 146S antigens of FMDV were the most stable when they were in TK with SLA. Although SLA improved the thermostability even when it was used with normal TBS, it was inferior to the sample suspended in TK with SLA (Figure 5).

## 4. Discussion

Since the Korean government set out to develop a domestic FMD vaccine after a large outbreak of FMD in 2010, FMDV O/SKR/JC/2014 (O JC), a local isolate, was initially selected as a candidate vaccine strain. However, the O JC strain was highly unstable and could not maintain structural integrity under general vaccine antigen production processes, including virus inactivation, during which other strains were barely affected. Among the seven serotypes of FMDV, serotypes O and SAT are, in particular, more unstable [19]. O JC, which belongs to the SEA topotype viruses (Mya-98 lineage) [20], appears to be the most unstable serotype O strain that we have previously dealt with. The O JC strain completely dissociated into 12S subunits by mild heating at 45 °C for 30 min, while other tested strains, including another serotype O virus, maintained over 80% of the initial content under the same conditions (Figure 1). In the current study, we introduced the O JC strain as a useful model virus for the stabilization study as its highly unstable feature enabled real-time efficient analyses of the stabilizing capacity of buffers and excipients.

In the storage test of 146S antigens, buffers that are frequently used in FMDV research were used. Among them, the TK buffer showed moderate preserving capacity as it could maintain over 60% of initial antigen content for up to 3 months in an aqueous phase at 4 °C (Figure 3a). Furthermore, 146S antigen recovery of the TK buffer in the frozen phase at −70 °C was the best among the tested buffers. However, the sole use of TK buffer without additional excipients resulted in a distinct reduction of antigen recovery, not only in the aqueous phase at 4 °C but also in the frozen phase at −70 °C as the storage duration increased (Figure 3).

Previously, Harmsen et al. reported that the combined use of 30% sucrose and 1% bovine serum albumin (BSA) in TK buffer was effective for the stabilization of FMDV during long-term storage at 4 °C when it was formulated into Marcol 52-based double oil emulsion vaccine, while 10% sucrose and 1% BSA combination was not [21]. However, if the 30% sucrose, which has high viscosity of 5.12 η at 4 °C [22], would be used for non-formulated vaccine antigens, it could burden the downstream processes, including filtration, as there is an inverse correlation between the flow rate of filtrate and liquid viscosity as Darcy’s law [23,24]. Moreover, BSA is a well-known allergen, whose residual content should not exceed 50 ng/dose in human vaccines [25,26]. In addition, in the veterinary field, several cases of allergic reactions after vaccination have been reported, and BSA was found to be one of the IgE-reactive vaccine components [27]. Lactalbumin hydrolysate, which was utilized in the present study, is known to be hypoallergenic as it is a complex of small peptides and amino acids, synthesized by enzymatic hydrolysis of lactalbumin [28].

Meanwhile, 20% sucrose (Suc), which was reported to increase the thermal stability of FMDV O/China/1999 [29], was still efficient in stabilizing the O JC vaccine antigen at 4 °C for 3 months; however, it did not show sufficient preservation afterward (Figure 4a). Although 10% sucrose with 5% lactalbumin hydrolysate (SLA) was already reported as effective for the long-term storage of freeze-dried FMD vaccine antigen at 4 °C [11], lyophilization itself is the most popular technique for the preservation of perishable materials such as proteins, particularly fit for the final product [30]. In this study, we found that SLA is also effective for the long-term preservation of the FMD vaccine antigen, not only in the undried frozen phase but also in the aqueous phase when it was used with TK buffer (Figure 4). A mixture of 5% dextran, 1% sodium glutamate, and 5% sucrose, which had shown similar effectivity with SLA in the long-term preservation of freeze-dried FMD vaccine antigen at 4 °C [11], was not tested further after the preliminary screening as it rather exacerbated the instability of O JC vaccine antigen in the aqueous phase 

As the pH of the solution highly affects not only the chemical integrity of a protein’s amino acid residues but also the maintenance of a protein’s higher-order structure, selection of the proper buffer is important for protein drugs [31], including the 146S antigen of FMDV, which is readily dissociated to 12S particles under mild acidic pH. According to a previous study, Tris-HCl buffer showed a slight elevation of pH from 7.37 at 25 °C to 8.54 at −30 °C, while sodium phosphate buffer exhibited a drastic decrease in pH from 7.0 at 25 °C to 3.36 at −30 °C [32]. Thus, using Tris-based buffers appeared to be more rational than phosphate buffers to maintain neutral to mild basic pH in both aqueous and frozen phases. Physiological salts, such as potassium chloride, have been used as tonicifying agents in protein formulations. In addition, chloride anions have been reported to selectively accumulate on the protein surface and directly influence the conformational integrity and stability of proteins more than cations [31,33]. Sucrose is known to stabilize proteins by preferential hydration in the liquid state as an osmolyte [31,34]. Lactalbumin hydrolysate is composed of various amino acids, including histidine, arginine, and glycine, which are also known to stabilize proteins through a variety of mechanisms such as preferential hydration, direct binding, buffering, and antioxidation [31,35,36]. Collectively, this individual stabilizing capacity of each component comprising SLA in TK buffer was expected to work synergistically.

In the present study, we validated that the SLA in TK buffer could preserve highly unstable FMDV O JC 146S antigens stably in both aqueous and frozen phases via real-time analyses for up to 1 year. Although the real-time analysis was only performed with the O JC strain, it was assumed that this stabilizer composition may fit for the other strains as they had no problem during the general production processes with even normal TBS. In the accelerated stability tests to confirm the wider applicability of the stabilizer composition to other strains, all tested strains showed the highest thermostability in the SLA in TK, although the sole use of the TK buffer or the SLA could mildly improve the thermostability of the tested FMDV (Figure 5).

## 5. Conclusions

Conclusively, adoption of the SLA in TK buffer for the FMD vaccine antigen preservation is valuable for the following reasons: first, it is advantageous for the phase change between the aqueous and frozen phases, which might be needed in case of the sudden halt of the production process; second, its components are generally regarded as safe in the viral vaccine field [37]; and finally, it allows a characteristically unstable FMDV strain to be dealt with like the other general strains.

Although further studies are required to decipher the underlying reason for the fragility of the O JC strain by determining which amino acid residues are involved in this stabilizing effect shown in a specific environment, this is the first study to identify the practical stabilizer composition that could be widely utilized not only in FMD research laboratories but also in FMD vaccine manufacturing facilities.

## Data Availability

Not applicable.
